# Liganded Thyroid Hormone Receptor Inhibits Phorbol 12-O-Tetradecanoate-13-Acetate-Induced Enhancer Activity via Firefly Luciferase cDNA

**DOI:** 10.1371/journal.pone.0028916

**Published:** 2012-01-13

**Authors:** Hiroko Misawa, Shigekazu Sasaki, Akio Matsushita, Kenji Ohba, Hiroyuki Iwaki, Hideyuki Matsunaga, Shingo Suzuki, Keiko Ishizuka, Yutaka Oki, Hirotoshi Nakamura

**Affiliations:** Second Division, Department of Internal Medicine, Hamamatsu University School of Medicine, Hamamatsu, Shizuoka, Japan; Institute of Genetics and Molecular and Cellular Biology, France

## Abstract

Thyroid hormone receptor (TR) belongs to the nuclear hormone receptor (NHR) superfamily and regulates the transcription of its target genes in a thyroid hormone (T3)-dependent manner. While the detail of transcriptional activation by T3 (positive regulation) has been clarified, the mechanism of T3-dependent repression (negative regulation) remains to be determined. In addition to naturally occurring negative regulations typically found for the thyrotropin β gene, T3-bound TR (T3/TR) is known to cause artificial negative regulation in reporter assays with cultured cells. For example, T3/TR inhibits the transcriptional activity of the reporter plasmids harboring AP-1 site derived from pUC/pBR322-related plasmid (pUC/AP-1). Artificial negative regulation has also been suggested in the reporter assay with firefly luciferase (FFL) gene. However, identification of the DNA sequence of the FFL gene using deletion analysis was not performed because negative regulation was evaluated by measuring the enzymatic activity of FFL protein. Thus, there remains the possibility that the inhibition by T3 is mediated via a DNA sequence other than FFL cDNA, for instance, pUC/AP-1 site in plasmid backbone. To investigate the function of FFL cDNA as a transcriptional regulatory sequence, we generated pBL-FFL-CAT5 by ligating FFL cDNA in the 5' upstream region to heterologous thymidine kinase promoter in pBL-CAT5, a chloramphenicol acetyl transferase (CAT)-based reporter gene, which lacks pUC/AP-1 site. In kidney-derived CV1 and choriocarcinoma-derived JEG3 cells, pBL-FFL-CAT5, but not pBL-CAT5, was strongly activated by a protein kinase C activator, phorbol 12-O-tetradecanoate-13-acetate (TPA). TPA-induced activity of pBL-FFL-CAT5 was negatively regulated by T3/TR. Mutation of nt. 626/640 in FFL cDNA attenuated the TPA-induced activation and concomitantly abolished the T3-dependent repression. Our data demonstrate that FFL cDNA sequence mediates the TPA-induced transcriptional activity, which is inhibited by T3/TR.

## Introduction

Thyroid hormone receptor (TR) belongs to the nuclear hormone receptor (NHR) superfamily. In a T3-dependent manner, TR not only activates (positive regulation) but also inhibits (negative regulation) its target genes. In contrast to that for the positive regulation, the molecular mechanism of the negative regulation has been elusive [1]–[4]. The thyrotropin β subunit (TSHβ) gene is a typical gene for which the expression is inhibited by T3-bound TR (T3/TR). Using deletion study of its promoter, a short DNA sequence, GGGTCA, immediately downstream of the transcription start site has been postulated as a negative T3 responsive element (nTRE). However, our detailed analysis in the presence of the transcription factors Pit1 and GATA2, which are the determinants of thyrotroph differentiation in pituitary gland [5], revealed that the reported nTRE is not necessary for T3-induced inhibition [6]. We proposed that the interference of GATA2-dependent transactivation by T3/TR is the main mechanism of negative regulation of the TSHβ gene by T3 [6]. In this scenario, T3/TR interacts with GATA2 but not DNA. This kind of inhibitory mechanism is referred to as “tethering”, where the transactivation function of DNA-binding transcription factor is suppressed by liganded NHR via protein-protein interaction [7]–[9]. For instance, AP-1-induced activity is repressed by T3/TR [10], [11] and liganded glucocorticoid receptor (GR) [7], [10], [12], while the activity of NFkB is inhibited by liganded GR and estrogen receptor (ER) α [9], [13], [14]. Ligand/receptor specificity was reported in the inhibition via tethering mechanism [6], [14] although direct DNA binding of NHRs is not required.

Unexpectedly, Lopez et al. [10] reported that a sequence, TGACACA, in pUC series plasmids mimics the function of AP-1 site (pUC/AP-1) and artificially mediates the negative regulation by T3/TR in Hela cells. pUC/AP-1 is included in the sequence, nt. 1/138, in pUC18/19 vector [15]. Because of the high yield in E. coli culture, pUC plasmid-derived backbone has been preferred in many plasmid constructions. pUC/AP-1 sequence is also found in pBR322 [16], which was the most common plasmid used for DNA cloning in the early period of molecular biology. pUC and pBR322-derived DNA fragments containing pUC/AP-1 sequence have been widely used as parts of so-called “homemade plasmids” as well as commercial plasmids, including some pGEM series (Promega, WI, U.S.A.). More than 2000 plasmid constructs bearing the sequence identical to nt. 1/138 in pUC18/19 vector were detected in the BLAST database. pUC- or pBR322-derived plasmid backbone was also utilized in the construction of reporter genes for the study of the negative regulation of the TSHβ gene by T3/TR [17]–[19].

Besides the negative regulation via pUC/AP-1 site, it was suggested that T3/TR may artificially suppress the activity of firefly luciferase (FFL)-based reporter genes driven by thymidine kinase (TK) promoter [20]–[22] or O-tet-CMV promoter [23]. These studies indicated artificial negative regulation via FFL cDNA in various cell lines. However, deletion analysis of FFL cDNA for the identification of the responsible sequence was not conducted since the negative regulation was evaluated by measuring the FFL enzymatic activity [20]–[23]. Thus, there remains the possibility that inhibition by T3 is mediated via a DNA sequence other than FFL cDNA in plasmid backbone, for example, pUC/AP-1 site.

It is noteworthy that artificial repression by T3/TR was not detected for chloramphenicol acetyl transferase (CAT)-based reporter gene in CV1 [20] and JEG3 cells [22], suggesting that CAT cDNA is safe, at least in these cell lines. We decided to investigate the function of FFL cDNA as a transcription regulatory sequence using CAT-based reporter gene. We generated pBL-FFL-CAT5 by fusing FFL cDNA to the upstream flanking region of the TK promoter in a CAT-based reporter plasmid, pBL-CAT5 [24], which lacks pUC/AP-1 site [10]. We found that FFL cDNA contains the sequence that is strongly activated by a protein kinase C activator, phorbol 12-O-tetradecanoate-13-acetate (TPA), in CV1 and JEG3 cells and that this activity is repressed by T3/TR. Current results support the previous reports that FFL cDNA mediates the artificial negative regulation by T3 [20], [22], [23].

## Materials and Methods

### Plasmid construction

**Figure 1:**
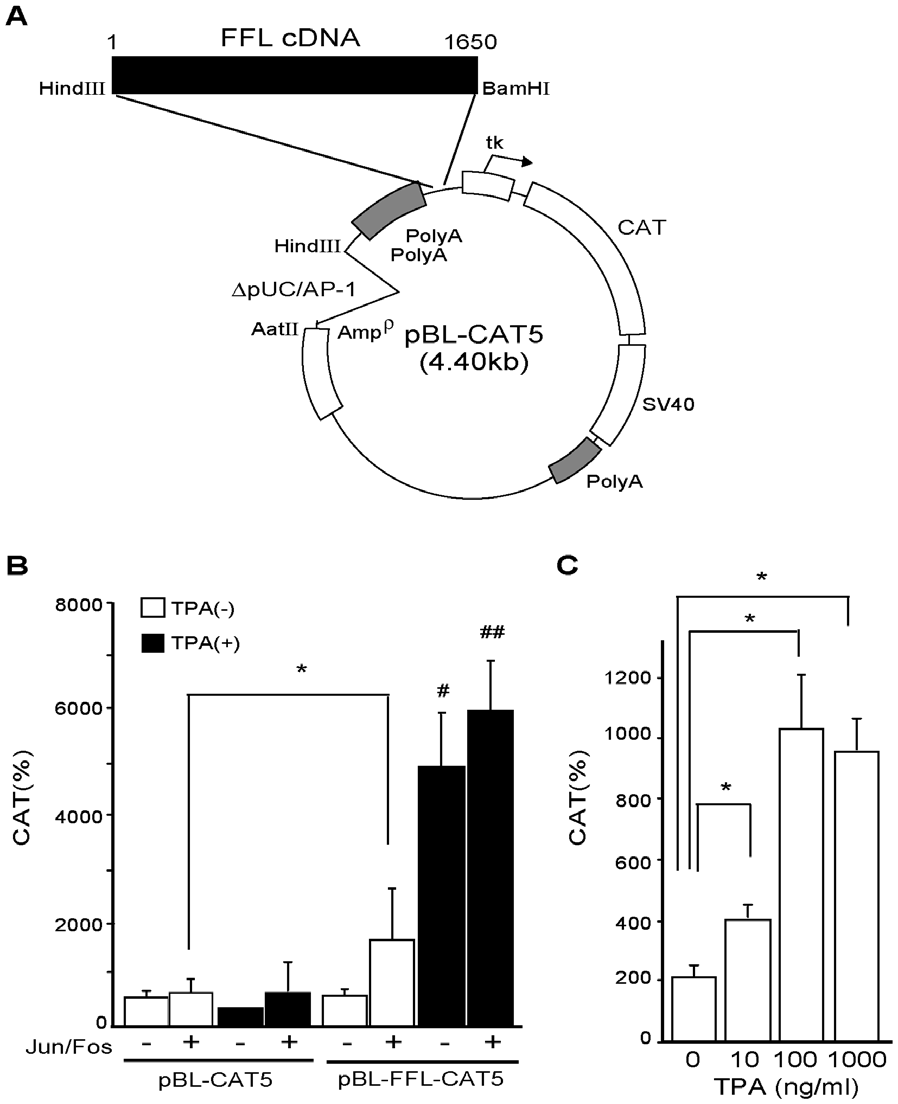
FFL cDNA functions as the TPA-induced enhancer in CV1 cells. A. Structure of pBL-FFL-CAT5. FFL cDNA was inserted into the HindIII-BamHI site in pBL-CAT5 (4.40 kb). For pBL-CAT5 construction, AatII-HindIII fragment of pBL-CAT2 [69] that contains pUC/AP-1 site was deleted and substituted by two fragments from SV40-poly-adenylation signals (poly-A) [24]. B. pBL-FFL-CAT5 but not pBL-CAT5 (mother plasmid) was strongly stimulated by TPA while activation by co-transfection of Jun and Fos was moderate. CV1 cells were transfected with 1.0 µg of the pBL-FFL-CAT5 with or without the expression vector for Jun and Fos (0.2 µg each). After incubation for 24 h in the presence or absence of 100 ng/ml TPA, CAT activity was measured with normalization of the transfection efficiency by β-galactosidase activity. The results are the means +/− SD from three independent experiments. *, P<0.05. #, the statistical significance (P<0.05) of TPA(−) vs. TPA(+). C. Transcriptional activity of pBL-FFL-CAT5 stimulated in a TPA dose-dependent manner. CV1 cells were transfected with 1.0 µg of the pBL-FFL-CAT5 in the presence of TPA (0–1000 ng/ml). The results are the means +/− SD from three independent experiments. *, P<0.05.

To isolate the DNA fragment containing the FFL coding region, the sequence GGATCT (nt. 1891/1896) at the 3′ flanking region of FFL cDNA in pGL2-basic vector (Promega, Madison, WI, U.S.A.) was mutated to the BamHI site (GGATCC) using a site-directed mutagenesis kit (Stratagene, La Jolla, CA, U.S.A.). This mutant plasmid was digested with HindIII and BamHI, and the 1750 bp DNA fragment was separated in gel electrophoresis and was ligated in the HindIII-BamHI site in the multi-cloning site in pBL-CAT5 [24] (Fig. 1A). Using the site-directed mutagenesis kit (Stratagene, U.S.A.), the positive TRE (pTRE)-like sequence (AGGTGA-CGCG-TGTGGCC) in the thymidine kinase (TK) promoter [25] of pBL-FFL-CAT5 was mutated to generate pBL-FFL-CAT5-mtk. Deletion mutants of pBL-FFL-CAT5 were generated using PCR and standard molecular biology techniques. The sequence between nt. 626/640 in FFL cDNA was mutated using the site-directed mutagenesis kit to generate the constructs, MA, MB, MC, and MD. The coding regions for hRluc and Luc2 were excised with HindIII and XbaI from phRL-null vector and pGL4.10 [luc2] vector (Promega), respectively. These DNA fragments were subcloned into the HindIII-XbaI sequence of pBL-CAT5 to create pBL-hRluc-CAT5 and pBL-Luc2-CAT5. All plasmid constructs were confirmed by sequencing. The expression plasmids for human TRβ1 (pCMX-hTRβ1), estrogen receptor α (pSG5-hERα), vitamin D3 receptor (pSG5-hVDR), retinoic acid receptor (RAR) α (pCMX-hRARα), and glucocorticoid receptor (GR) (pCMX-hGR) were as described previously [26], [27].

### Cell culture and transient transfection

A monkey kidney cell line, CV1 [28], and a human embryonic kidney cell line, 293T [29], were grown in monolayer culture at 37C under 5% CO_2_/95% air in Dulbecco's modified Eagle's medium (DMEM) containing 10% fetal calf serum, penicillin G (100 units/ml), and streptomycin (100 µg/ml). CV1 and 293T cells were trypsinized and plated in 60-mm dishes for 24 h before transient transfection using the calcium-phosphate technique [30]. Cells at a density of 10^6^ cells/plate were transfected with 0.4 µg of TR expression vector (pCMX-hTRβ1) along with 1.0 µg of pBL-FFL-CAT5, 1.8 µg of β-galactosidase expression vector pCH111 (a modified version of pCH110; Pharmacia LKB Biotechnology, Piscataway, NJ, U.S.A.), and pCMX empty vector as carrier DNA (to adjust the total to 5.2 µg of DNA/dish). After the cells were exposed to the calcium phosphate/DNA precipitates for 20 h, the medium was replaced with fresh DMEM containing 5% fetal calf serum depleted of thyroid hormones or the same medium was supplemented with 100 ng/ml phorbol 12-O-tetradecanoate-13-acetate (TPA) and/or 1 µM T3. After incubation for an additional 24 h, the cells were harvested and the CAT activity was measured and normalized by the β-galactosidase activity as described previously. JEG3 cells [31] were cultured in OptiMEM-1 medium (BRL-Gibco, Grand Island, NY, USA) containing 2% fetal calf serum, penicillin G (100 units/ml), and streptomycin (100 µg/ml). The cells were trypsinized 12–18 h before transfection and plated into medium containing 2% T3-stripped fetal calf serum. The cells were transfected with 1 µg of pBL-FFL-CAT5, 1.8 µg of pCH111, 0.4 µg of pCMX-hTRβ1, and pCMX empty vector as carrier DNA to adjust the total to 5.2 µg of DNA/dish [32]. After 4 h of exposure to calcium phosphate/DNA precipitates, the cells were cultured in medium with 1 µM T3 and/or 100 ng/ml TPA for 36–48 h and the CAT and β-galactosidase activities were measured. In CAT reporter assay, we also performed transfection with CMV (cytomegalovirus)-CAT (10 ng/dish), the magnitude of which was adjusted to 100%.

### Gel shift assay

FFL14 probe (sense: 5′-CCTAAGGGTGTGGCCCTTCCGCATAGAACTGCCTGCGTCA-3′ and antisense: 5′-TGACGCAGGCAGTTCTATGCGGAAGGGCCACACCCTTAGG-3′) and consensus AP-1 probes (sense: 5′-TCGACGGTATCGATAAGCTATGACTCATCCGGGGGATC-3′ and antisense: 5′-GATCCCCCGGATGAGTCATAGCTTATCGATACCGTCGA-3′) were annealed and labeled with γ-^32^P-ATP using T4 polynucleotide kinase (Toyobo, Tokyo, Japan). CV1 nuclear extracts (CV1-NE) were prepared as described previously [33]. The γ-^32^P-labeled probes and 2 µg of CV1-NE were incubated for 30 min on ice in 20 µl of binding buffer containing 20 mM Hepes-NaOH (pH 7.9), 50 mM KCl, 0.05 mM EDTA, 2.5 mM MgCl_2_, 8.5% glycerol, 1 mM dithiothreitol, and 0.5 µg/ml poly(dI-dC). For the supershift assays, anti-cJun antibody (no. sc-1694x, Santa Cruz Biotechnology, CA) was added to the binding reaction mixture. DNA-protein complexes were resolved by electrophoresis on a 5% polyacrylamide gel at 150 V for 120 min at room temperature. For the competition assay, 100-fold excess of cold FFL14G was added. The gel was dried and labeled bands were visualized using the BAS-1000 autoradiography system (Fuji Film, Tokyo, Japan).

## Results

### FFL cDNA contains the sequence that is activated by TPA

Because it was reported that CAT-based reporter genes do not mediate the repressive effect by T3/TR [20], [22], we selected CAT reporter system to study whether FFL cDNA possesses the sequence mediating the inhibition by T3/TR. A CAT reporter gene, pBL-CAT5 plasmid (Fig. 1A), was utilized because reported pUC/AP-1 site, TGACACA, was deleted [24]. Moreover, in pBL-CAT5, two poly-A signals of SV40 large T encoding gene were inserted into the 5′ region of the multi-cloning sites to abrogate the influence of upstream signal. We ligated the FFL cDNA in the pBL-CAT5, creating pBL-FFL-CAT5 (Fig. 1A). We employed a monkey kidney-derived cell line, CV1 [28], because it has been widely used in the study of T3-dependent positive and negative regulations [6], [20], [26], [34]–[37]. On the basis of the speculation that FFL cDNA may contain the sequence responsive to PKC signaling as in the case of pUC/AP-1 site [10], we tested the effect of TPA on the activity of FFL cDNA fused with TK promoter. As shown in Fig. 1B, pBL-FFL-CAT5 was robustly stimulated by TPA treatment while moderately activated by co-transfection of Jun and Fos. The transactivation of pBL-FFL-CAT5 by TPA occurred in a dose-dependent fashion (Fig. 1C). In contrast, pBL-CAT5 was not activated by co-transfection of Jun and Fos or TPA treatment (Fig. 1B), suggesting that pBL-CAT5 does not have the cryptic sequence that mimics AP-1.

### TPA-induced transactivation of pBL-FFL-CAT5 is suppressed by T3/TR

**Figure 2:**
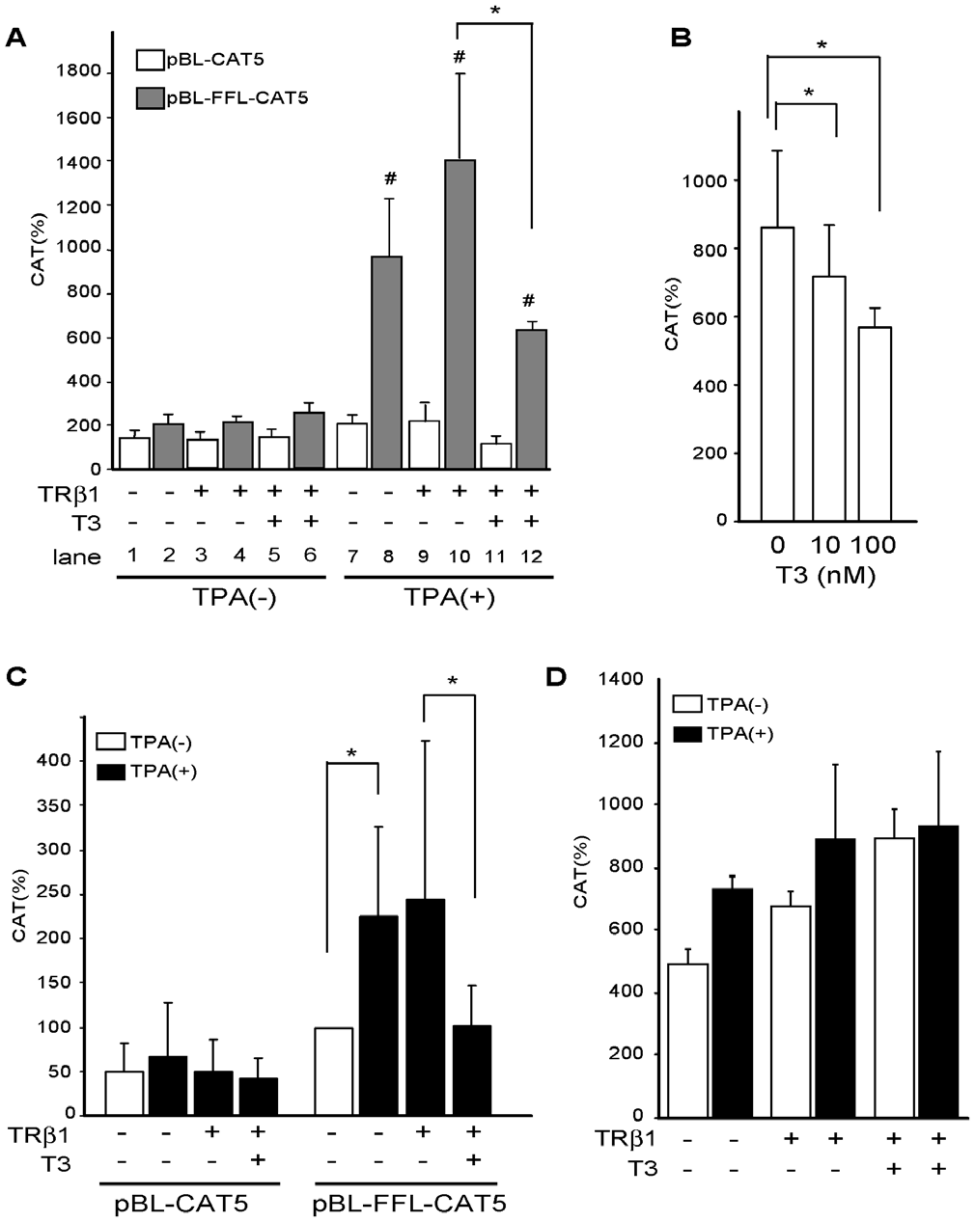
T3/TR inhibits the transcriptional activity of FFL cDNA stimulated by TPA in CV1 and JEG3 but not 293T cells. A. T3/TR inhibits the transcriptional activity of pBL-FFL-CAT5 but not pBL-CAT5 stimulated by TPA in CV1 cells. TRβ1 expression vector (0.4 µg) was transfected into CV1 cells along with 1.0 µg of the pBL-FFL-CAT5 or pBL-CAT5. After incubation for 24 h in the presence or absence of 100 ng/ml TPA and/or 1 µM T3, CAT activity was measured with normalization of the transfection efficiency by β-galactosidase activity. The results are the means +/− SD from four independent experiments. *, P<0.05. #, the statistical significance (P<0.05) of TPA(−) vs. TPA(+). B. T3 dose dependence in the inhibition of pBL-FFL-CAT5 activated by TPA. pBL-FFL-CAT5 was transfected into CV1 cells in the presence of 100 ng/ml TPA and 0–100 nM T3. C. T3/TR inhibited the transcriptional activity of pBL-FFL-CAT5, but not pBL-CAT5, stimulated by TPA in JEG3 cells. TRβ1 expression vector (0.4 µg) was transfected into JEG3 cells along with 1.0 µg of pBL-FFL-CAT5 or pBL-CAT5 in the presence or absence of 100 ng/ml TPA and/or 1 µM T3. The results are the means +/− SD from four independent experiments. *, P<0.05. D. Enhancer activity of FFL cDNA is not activated by TPA or inhibited by T3/TR in 293T cells. TRβ1 expression vector (0.4 µg) was transfected into 293T cells along with 1.0 µg of pBL-FFL-CAT5 or pBL-CAT5. After incubation for 24 h in the presence or absence of 100 ng/ml TPA and/or 1 µM T3, CAT activity was measured with normalization of the transfection efficiency by β-galactosidase activity. The results are the means +/− SD from three independent experiments. *, P<0.05.

As shown in Fig. 2A and B, TPA-induced transactivation of pBL-FFL-CAT5 was repressed by T3/TR. We examined the effect of other liganded NHRs on the TPA-dependent transcriptional activity of pBL-FFL-CAT5 (data not shown). There was no significant effect on the TPA-induced transcriptional activity by liganded-ERα, vitamin D3 receptor, and retinoic acid receptor α. Thus, there is receptor/ligand-specificity in the FFL cDNA-mediated inhibition. Unexpectedly, this activity was not repressed by the liganded GR, suggesting that the transcription factor mediating TPA signal may not be Jun/Fos [7], [10], [12]. Human choriocarcinoma cell line, JEG3 [31], has been utilized for the analysis of T3-dependent negative regulation [32], [38]–[45]. In this cell line, pBL-FFL-CAT5 was also activated by TPA and inhibited by T3/TR (Fig. 2C). In contrast, no TPA-induced activation or negative regulation by T3/TR was detected in human embryonic kidney-derived 293T cells [29] (Fig. 2D), indicating that this cell line lacks the transcription factor conducting TPA signal to FFL cDNA sequence.

### Reported half-site-like sequences in the TK promoter are not involved in the negative regulation of the TPA-induced activation by T3

**Figure 3:**
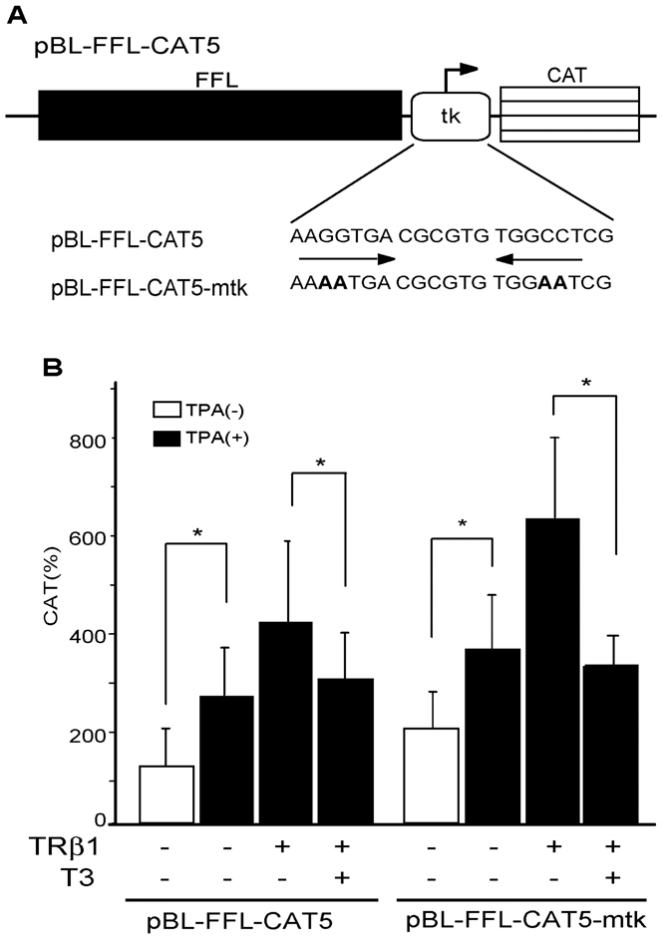
TPA-induced activation or the negative regulation by T3 was not affected by the reported pTRE (half-site-like sequences) in the TK promoter. A. Schematic representation of pBL-FFL-CAT5 (WT) and its mutant, pBL-FFL-CAT5-mtk. The nucleotides in both half-sites (arrows) in the reported palindromic TRE in TK promoter [25] were mutated in pBL-FFL-CAT5-mtk (boldface). B. The mutation of reported pTRE does not affect the TPA-induced transcriptional activity or the repression by T3/TR. In the presence or absence of TPA (100 ng/ml) and or T3 (1 µM), pBL-FFL-CAT5 (WT) or pBL-FFL-CAT5-mtk was transfected into CV1 cells with or without TRβ1 expression plasmid. CAT activity was measured with normalization of the transfection efficiency by the β-galactosidase activity. The results are the means +/− SD from three independent experiments. *, P<0.05.

In the positive regulation, TR heterodimerizes with retinoid X receptor on positive T3-responsive element (pTRE), which consists of a direct repeat of half-sites (typically, AGGTCA) with 4 random spacing nucleotides (direct repeat 4, DR4) [34]. Tillman et al. [20] and Maia et al. [22] suggested the possibility that, when strong pTRE exists in the promoter, T3-induced activation may mask the inhibitory effect by T3 on the transcriptional activity via FFL cDNA. According to Park et al. [25], the thymidine kinase (TK) promoter contains a pTRE-like sequence (nt. -22/-2, AGGTGA-CGCGTG-TGGCCT, Fig. 3A), which may confer T3-dependent activation in COS [46], GC [47], and GH4C1 cells [25]. However, as shown in Fig. 2A (lanes 3 and 5), T3/TR did not activate this promoter in pBL-CAT5 in CV1 cells. To exclude another possibility that one of these half-site-like sequences functions as nTRE, we generated pLB-FFL-CAT5-mtk, in which the DNA sequences of both half-sites were mutated (Fig. 3A). As shown in Fig. 3B, both TPA-induced activation and inhibition by T3/TR were maintained. These results indicated that the reported pTRE or the half-site-like sequences in the TK promoter do not affect the T3/TR-dependent negative regulation in CV1 cells.

### FFL cDNA contains the sequence that mediates TPA-induced activation

**Figure 4:**
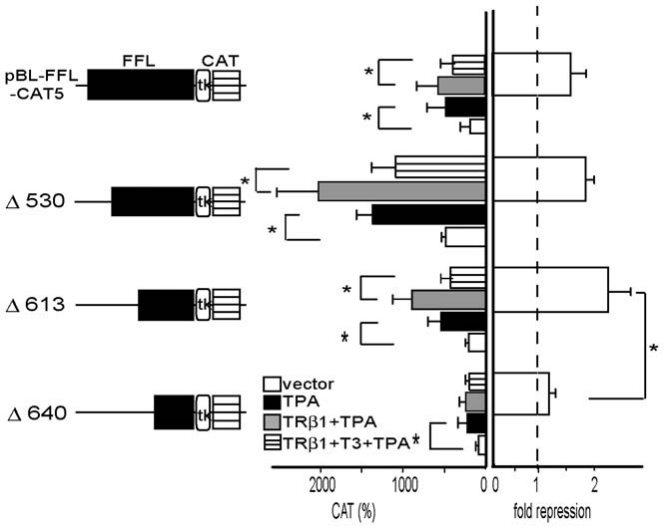
Deletion analysis of FFL cDNA as a transcriptional regulatory sequence. Schematic representation of deletion mutants of pBL-FFL-CAT5 is illustrated in the left panel. Fold repression (right panel) was calculated from CAT activity without T3 divided by that with T3. In the deletion mutant Δ640, basal activity was reduced. Although TPA treatment modestly but significantly potentiated the promoter activity, repression by T3/TR was blunted (middle and right panels). CAT activity was measured with normalization of the transfection efficiency by β-galactosidase activity. The results are the means +/− SD from three independent experiments. *, P<0.05.

nTRE was defined as the sequence where unliganded TR may function as a transcriptional activator [17]. However, the existence of nTRE is unlikely because unliganded TR alone did not activate pBL-FFL-CAT5 in CV1 cells (Fig. 2A, lanes 2 and 4). We speculated on the involvement of a tethering mechanism [7]–[9], where activity of TPA-dependent transcription factor is inhibited by T3/TR. We carried out deletion analysis of the FFL cDNA (Fig. 4, left panel). As shown in Fig. 4 (middle panel), the deletion of nt. 1/530 increased the basal and the TPA-induced activities while truncations of nt. 531/613 and nt. 614/640 reduced them. These results suggest the existence of the inhibitory sequence(s) in nt. 1/530 and at least two activation sequences in nt. 531/613 and nt. 614/640. The finding that TPA treatment modestly but significantly potentiated the transcription of the Δ640 constructs suggested the existence of the weak TPA-responsive element(s) in the sequence downstream of nt. 640. Interestingly, negative regulation by T3/TR was blunted in the Δ640 construct (Fig. 4, right panel). These results suggest that the DNA sequence between nt. 613/640 may include the sequence responsible for the inhibition by T3/TR.

### The CV1-derived nuclear protein that recognizes the DNA sequence between nt. 626/640 is not AP-1

**Figure 5:**
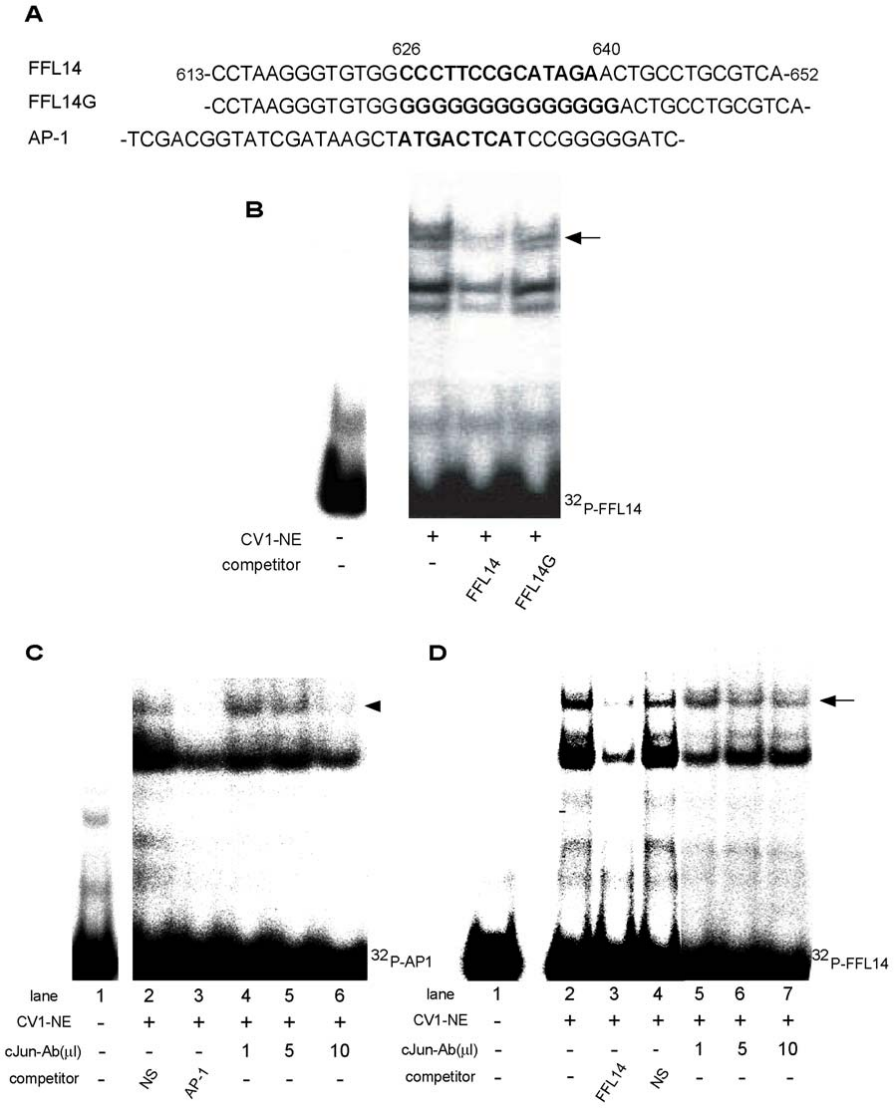
The CV1-derived nuclear protein that recognizes the DNA sequence between nt. 626/640 is not AP-1. A. Sense sequence of FFL14 oligo DNA probe encompassing nt. 613/652, its mutant (FFL14G), and AP-1 oligo DNA probe [70]. The sequence that has weak homology with AP-1 site in FFL14 oligo and consensus Jun/Fos binding sequence in AP-1 are indicated in boldface. FFL14G that has mutations in the sequence similar to AP-1 site was used as control for competition assay. B. Gel shift assay using radiolabeled FFL14 with CV1 nuclear extract (CV1-NE). Arrow indicates the specific binding. C. Gel shift assay using radiolabeled AP-1 probe [70] with CV1-NE. Arrowhead indicates the specific binding containing cJun. The binding signal was abolished by the addition of anti-cJun antibody (lanes 5 and 6). D. Gel shift assay using radiolabeled FFL14 probe with CV1-NE in the presence of anti-cJun antibody. The binding signal (arrow) was not affected by the addition of anti-cJun antibody (lanes 5–7). NS, non-specific cold competitor.

Using nuclear extract from CV1 cells (CV1-NE), we performed gel shift assay with ^32^P-labeled DNA probes encompassing various regions between nt. 613/640. The sequence between nt. 626/640 has weak homology with consensus AP-1 site (Fig. 5A). We found the specific binding signal at nt. 626/640 (Fig. 5B) that was competed with by the 100-fold molar excess of the cold specific competitor, but not its mutant (FFL14G) (Fig. 5B). As a control experiment, we performed gel shift assay using CV1-NE and ^32^P-labeled consensus AP-1 site (Fig. 5A). As shown in Fig. 5C, we detected the binding signal with consensus AP-1 site. This signal was specifically abolished by the 100-fold molar excess of the cold specific competitor and eliminated by anti-cJun antibody. These results indicated the existence of endogenous cJun in CV1 cells. However, as shown in Fig. 5D, the binding signal with FFL14 was not affected by the addition of anti-cJun antibody, suggesting that AP-1 is not the nuclear protein recognizing the sequence between nt. 626/640 in CV1 cells.

### The DNA sequence between nt. 626/640 in FFL cDNA mediates both TPA-induced activity and negative regulation by T3/TR

**Figure 6:**
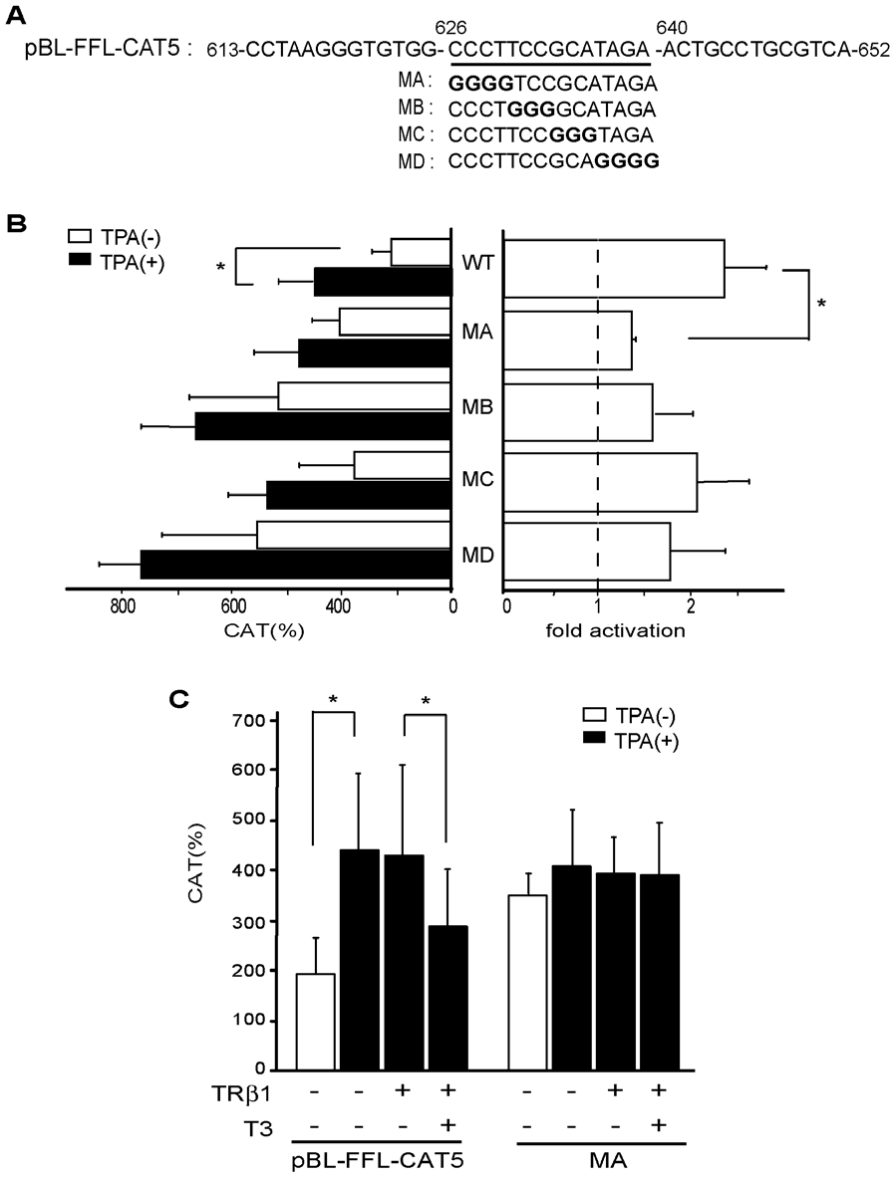
The sequence between nt. 626/640 mediates both TPA-dependent activation and the negative regulation by T3/TR. A. Schematic representation of pBL-FFL-CAT5 (WT) and its mutants, MA, MB, MC, and MD. B. The stimulatory effect by TPA is decreased in MA. In the presence or absence of TPA (100 ng/ml), pBL-FFL-CAT5 (WT) or its mutants (MA-D) were transfected into CV1 cells. CAT activity was measured with normalization of the transfection efficiency by the β-galactosidase activity (left panel). Fold activation (right panel) was calculated from CAT activity with TPA divided by that without TPA. C. The sequence between nt. 626/640 in the FFL cDNA mediates both TPA-dependent activation and negative regulation by T3/TR. In the presence or absence of TPA (100 ng/ml) and/or T3 (1 µM), pBL-FFL-CAT5 (WT) or its mutant, MA, was transfected into CV1 cells with or without TRβ1 expression plasmid. CAT activity was measured with normalization of the transfection efficiency by the β-galactosidase activity. The results are the means +/− SD from three independent experiments. *, P<0.05.

On the basis of the results of the gel shift assay in Fig. 5, we generated the scanning mutations between nt. 626/640 (Fig. 6A). As shown in Fig. 6B (left panel), the basal transcriptional activities without TPA were increased in these mutants, suggesting that the sequence between nt. 626/640 has inhibitory function. We found that TPA-induced stimulation evaluated by fold activation was significantly reduced in the mutant MA (Fig. 6B, right panel). The activity of the putative TPA-response sequence(s) downstream of nt. 640 was thought to be masked in this mutant (Fig. 6B and C) presumably because it is very weak (Fig. 4, middle panel). Importantly, the repression by T3/TR was not detected in MA (Fig. 6C), suggesting that the DNA sequence between nt. 626/640 mediates not only the TPA-dependent activation but also the artificial negative regulation by T3/TR.

### cDNAs of modified luciferase, hRluc, and Luc2 do not mediate TPA-induced activation or negative regulation by T3/TR in CV1 cells

**Figure 7:**
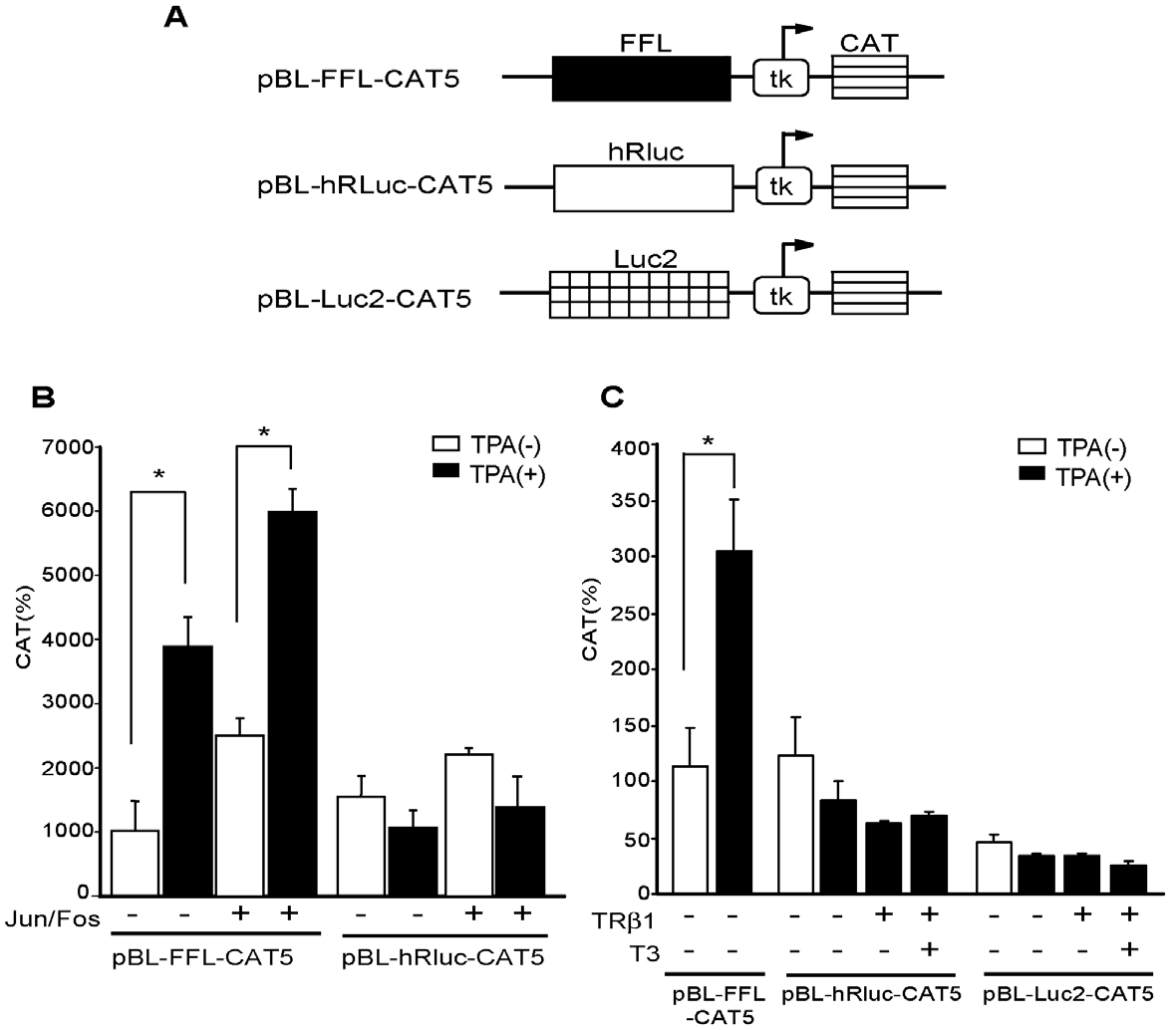
cDNAs of modified luciferase, hRluc and Luc2, do not mediate TPA-induced activation or negative regulation by T3/TR in CV1 cells. A. Schematic representation of pBL-FFL-, pBL-hRluc-, and pBL-Luc2-CAT5. The cDNA of FFL, hRluc, or Luc2 was subcloned into the multi-cloning site of pBL-CAT5, which lacks pUC/AP-1 site. B. Co-transfection of Jun/Fos and/or treatment of TPA potentiated the enhancer activity of FFL cDNA, but not hRluc cDNA. pBL-FFL-CAT5 or pBL-hRluc-CAT5 (1.0 µg) was transfected into CV1 cells with or without the expression plasmids for Jun and Fos (0.2 µg each). After incubation for 24 h in the presence or absence of 100 ng/ml TPA, CAT activity was measured with normalization of the transfection efficiency by β-galactosidase activity. The results are the means +/− SD from three independent experiments. *, P<0.05. C. The cDNAs of hRluc [48] and Luc2 [51] do not function as TPA-induced enhancers and are not affected by T3/TR. TRβ1 expression vector (0.4 µg) was transfected into CV1 cells along with 1.0 µg of the pBL-FFL-, pBL-hRluc-, or pBL-Luc2-CAT5. After incubation for 24 h in the presence or absence of 100 ng/ml TPA and/or 1 µM T3, CAT activity was measured with normalization of the transfection efficiency by β-galactosidase activity. The results are the means +/− SD from three independent experiments. *, P<0.05.

The sequence homology between FFL and modified Renilla reniformis-derived luciferase (hRluc) [48] is low (9.8% at amino acid level, 8.4% at DNA level) and there is no sequence in hRluc-cDNA similar to nt. 613/640 of FFL-cDNA. To confirm that hRluc cDNA does not respond to TPA stimulation, we fused hRluc cDNA to the pBL-CAT5, generating pBL-hRluc-CAT5 (Fig. 7A). pBL-hRluc-CAT5 was not activated by TPA or Jun/Fos co-expression in CV1 cells (Fig. 7B) and its activity was not affected by T3/TR (Fig. 7C). To eliminate the artificial transactivation mediated by the sequence of cDNA of FFL [49], [50] or Luc+ [51], modified luciferase, Luc2, was also developed [51]. For the evaluation of Luc2 cDNA, we generated pBL-Luc2-CAT5, in which Luc2 cDNA was ligated in pBL-CAT5 (Fig. 7A). Again, pBL-Luc2-CAT5 was not activated by TPA or suppressed by T3/TR (Fig. 7C).

## Discussion

In this study, we confirmed the existence of the sequence in FFL cDNA that mediates artificial negative regulation by T3/TR. FFL assay has many advantages compared with CAT assay [52], [53]. First, FFL assay is more sensitive and has broader linearity than CAT assay. Second, no radioactivity is required and the assay procedure is very quick and safe. Finally, FFL assay is more appropriate for kinetic study than CAT assay because, in mammalian cells, the half-life of FFL protein is much shorter (approx. 3 hours) than that of CAT protein (approx. 50 hours). By virtue of these advantages, FFL-based reporter assays have been utilized in numerous studies for transcriptional regulation. For example, when PubMed database was searched with the keywords of “phorbol” and “luciferase”, 836 publications were retrieved (in April, 2011). FFL-based reporter assays were also employed in studies of T3-induced negative regulation [35]–[37], [40], [43], [54]–[59]. However, we demonstrated here that, in CV1 [28] and JEG3 cells [31], the FFL cDNA functions as TPA-induced enhancer (Fig. 1B and C) and its activity is inhibited by T3/TR (Fig. 2A, B and C). There are at least two activation sequences in FFL cDNA. One is located downstream of nt. 640 (Fig. 4) and another is between nt. 626/640 (Fig. 6C). The latter may be an inhibitory sequence because transcriptional activity was increased by its mutation (Fig. 6B, left panel) and this inhibition was relieved by the TPA treatment (Fig. 6C). The TPA-responsive sequence downstream of nt. 640 appears to be much weaker than that at nt. 626/640. In agreement with the findings in Fig. 4 (right panel), the results in Fig. 6C (right panel) suggest that the sequence between nt. 626/640 (Fig. 4) also mediates the negative regulation by T3/TR in CV1 cells. We speculate that the negative regulation via FFL cDNA is mediated by a tethering mechanism [7]–[9], but not so-called nTRE [17]. First, when the sequence nt. 626/640 was mutated (Fig. 6A), the negative regulation of FFL cDNA was abolished concomitantly with TPA-induced activity (Fig. 6C). Second, there is no sequence between nt. 626/640 that has homology with half-site. Third, although nTRE was previously defined as the DNA sequence on which unliganded TR may function as a transcriptional activator [17], the current results rule out the presence of this kind of DNA sequence in FFL cDNA (Fig. 2A, lanes 2 and 4). Finally, because CV1 cells do not have endogenous TR [34], the protein that binds with the sequence between nt. 626/640 (Fig. 5B and D) should not be TR.

Although one may argue that, in experimental conditions without PKC activity or Jun/Fos, FFL-based reporter assay can be utilized for the study of negative regulation, at least 11 different PKC subtypes have been reported and some of them are expressed ubiquitously [60]. Moreover, PKC activates multiple transcription factors besides Jun/Fos. For example, the major transcription factor that mediates TPA signal via FFL cDNA in CV1 cells is not Jun/Fos (Fig. 5). The length of FFL cDNA (1653 bp) is much longer than that of CAT gene (657 bp). Computer search predicts more than 250 potential sites for DNA binding transcription factors in FFL cDNA [50]. The cDNAs of modified luciferase, Luc+ [51], [61], and conventional Renilla luciferase [48], [62], [63] also harbor numerous short sequences that can be recognized by a variety of transcription factors. It may be useful to search known binding sites in the reporter gene [50]. We also recommend computer search for pUC/AP-1 site (TGACACA) [10]. However, the information of the full-length sequence is not always available in “home-made plasmids”. Involvement of a transcription factor is usually difficult to predict owing to redundancies of the transcription factor binding sites. Modified luciferase genes including hRluc [48] and Luc2 [51] may be safer than FFL (Fig. 7B and C) presumably because the majority of predicted transcription factor binding sites were mutated. To verify the effect of various stimulations or chemical compounds in different cell lines, reporter assays with pBL-FFL-, hRluc-, and Luc2-CAT5 may be informative.

Although the artificial negative regulation by FFL cDNA or pUC/AP-1 site can be neglected when the promoter/enhancer activity is much stronger than that of these sequences [20], it should be kept in mind that deletion or mutation analyses of promoters often decrease their transcriptional activities. Once the activity of the deleted promoter becomes less than that of FFL cDNA or pUC/AP-1, the reporter signal may be artificially maintained by these sequences, which can be reduced by T3/TR. Madison et al. [40] performed deletion analysis of the α-glycoprotein subunit (GSU) promoter in JEG3 cells to identify its nTRE. Their FFL assay showed that negative regulation by T3/TR was maintained even in the minimal promoter containing only TATA box (nt. −35). There is the possibility that the activity of FFL cDNA surpassed that of minimal αGSU promoter (TATA box) and caused artificial negative regulation. A similar problem was suggested in a study that attempted to define the structure of nTRE. Using an FFL-based reporter gene, Näär et al. [35] reported that deletion of spacing nucleotides of DR4-type TRE converted T3/TR from transcriptional activator to repressor and proposed that unspaced direct repeat (DR0) might be an nTRE. However, their observation was not reproduced with a CAT-based construct (private communication from Kazuhiko Umesono). Tillman et al. [20] speculated that FFL cDNA-mediated negative regulation might be regenerated after the disruption of DR4-type pTRE by deleting spacing nucleotides.

Negative regulation has been regarded as the mirror image of positive regulation and unliganded TR was postulated to be a transcriptional activator. On the basis of this hypothesis, deletion analysis of the TSHβ gene was performed in the absence of T3 and the putative nTRE was reported [17]. Unexpectedly, however, TSHβ expression was not reduced in TR-null mice [64]. This finding suggests that unliganded TR per se is not necessary for the transcription of the TSHβ gene and that another transcription factor maintains the basal transcriptional activity before inhibition by T3. Indeed, transcriptional activity by unliganded TR was extremely low and almost negligible compared with that driven by Pit1 and GATA2 [6], [26], which are the essential activators of the TSHβ gene [5], [33]. Using CAT-based reporter gene lacking the pUC/AP-1 site, we carried out deletion analysis of the TSHβ promoter under the co-expression of GATA2 and Pit1. We found that the reported nTRE is not required for negative regulation by T3 [6]. We proposed a model in which the mechanism for the T3-dependent inhibition is mediated by the tethering of T3/TR with GATA2 [6]. Although the genes for αGSU, prepro-thyrotropin releasing hormone, and myosin heavy chain β are also inhibited by T3/TR and putative nTREs have been postulated in these genes, unliganded TRs may not be a transcriptional activator because their expression levels were not reduced in TR-null mice [57], [64]–[67].

Because the majority of previous studies paid little attention to the artificial inhibition by T3/TR via FFL cDNA or pUC/AP-1 site, there has remained the possibility that the transcriptional activity via FFL cDNA or pUC/AP-1 site might be regarded as that by unliganded TR. Current data combined with our previous reports [6], [26] suggest that the characterization of the mechanism for the basal transcriptional activity before T3 addition as well as the selection of reporter gene is very important. Although FFL assay has broad linearity, careful interpretation and appropriate control are necessary when the promoter activity before T3 addition decreases in the course of deletion analysis. Information on the factor required for the basal promoter activity is helpful to avoid the artificial negative regulation via the sequence in plasmid backbone. A strategy to study the negative regulation by T3/TR should be established in future [1]–[4] because approximately 20–50% of T3-sensitive genes are negatively regulated by T3/TR in vivo [4], [68] and it may also provide insight into the mechanism of ligand-dependent negative regulation by other NHRs.
